# A Combination of Deep-Sea Water and Fucoidan Alleviates T2DM through Modulation of Gut Microbiota and Metabolic Pathways

**DOI:** 10.3390/ph16030462

**Published:** 2023-03-20

**Authors:** Shan He, Wei-Bing Peng, Hong-Lei Zhou, Xian-Jun Fu, Yan-Hua Sun, Zhen-Guo Wang

**Affiliations:** 1 College of Pharmaceutical Science, Shandong University of Traditional Chinese Medicine, Jinan 250355, China; 2 Biology Institute, Qilu University of Technology (Shandong Academy of Sciences), Jinan 250103, China; 3 Institute for Literature and Culture of Chinese Medicine, Shandong University of Traditional Chinese Medicine, Jinan 250355, China

**Keywords:** deep-sea water, fucoidan, T2DM, fecal metabolomics, gut microbiota

## Abstract

Fucoidan and deep-sea water (DSW) are attractive marine resources for treating type 2 diabetes (T2DM). In this study, the regulation and mechanism associated with the co-administration of the two were first studied using T2DM rats, induced by a high fat diet (HFD) and streptozocin (STZ) injection. Results demonstrate that, compared to those with DSW or FPS alone, the orally administered combination of DSW and FPS (CDF), especially the high dose (H-CDF), could preferably inhibit weight loss, decrease levels of fasting blood glucose (FBG) and lipids, and improve hepatopancreatic pathology and the abnormal Akt/GSK-3β signaling pathway. The fecal metabolomics data show that H-CDF could regulate the abnormal levels of metabolites mainly through the regulation of linoleic acid (LA) metabolism, bile acid (BA) metabolism, and other related pathways. Moreover, H-CDF could adjust the diversity and richness of bacterial flora and enrich bacterial groups, such as *Lactobacillaceae* and *Ruminococcaceae UCG-014*. In addition, Spearman correlation analysis illustrated that the interaction between the gut microbiota and BAs plays an essential role in the action of H-CDF. In the ileum, H-CDF was verified to inhibit activation of the farnesoid X receptor (FXR)–fibroblast growth factor 15 (FGF15) pathway, which is regulated by the microbiota–BA–axis. In conclusion, H-CDF enriched *Lactobacillaceae* and *Ruminococcaceae UCG-014*, thereby changing BA metabolism, linoleic acid metabolism, and other related pathways, as well as enhancing insulin sensitivity and improving glucose and lipid metabolism.

## 1. Introduction

Across the globe, the morbidity of type 2 diabetes mellitus (T2DM) is rising year after year, and the number of patients diagnosed with T2DM is predicted to increase to 0.5 billion by 2025 [1]. Therefore, effective prevention and control measures are urgently needed. Fucoidan and deep-sea water (DSW) are marine resources that have attracted much attention in recent years. Fucoidan is a unique polysaccharide in brown algae, and numerous studies have suggested that fucoidan exerts multiple biological effects, such as hypoglycemic, lipid-lowering, anticoagulation, antitumor, and antiviral activities [2,3,4]. DSW refers to seawater more than 200 m deep [5]. DSW is rich in elements that promote human metabolism, especially magnesium (Mg) and beneficial trace elements, such as vanadium (V), chromium (Cr), zinc (Zn), and selenium (Se) [6]. More and more studies have demonstrated that DSW exerts various pharmacological effects, such as the prevention and treatment of hyperlipidemia [5] and T2DM [7,8], along with improvement of the immune system [9], anti-tumor properties [10], and treatment of osteoporosis [11]. In particular, it has shown promising effects in metabolic diseases [5,7,8,12,13].

Our previous study demonstrated that the combination of fucoidan and DSW could effectively modify glucolipid metabolism disorders in HepG2 cells to alleviate insulin resistance [14], but the mechanism of action is still unclear. Metabolomics can provide richer information on endogenous substances in the body, revealing the action mechanism of drugs at therapeutic levels [15]. Intestinal flora play significant roles in host health; moreover, the structure, composition, and metabolism can influence the pathogenesis and progression of T2DM [16]. Therefore, the relationship between microbiota imbalance and diabetes has attracted much attention [17]. In particular, fecal metabolomics is an integrated analysis of metabolomics and gut microbiota, which can effectively describe the relationship between intestinal flora and the host, offering more comprehensive information on the metabolic profile of the host [18].

In this research, we established a T2DM rat model, which was induced by a high-fat diet (HFD) plus streptozocin (STZ) injection, and observed the combined effect of DSW and fucoidan. Thereafter, fecal metabolomics, combined with 16S rDNA analysis, was applied to investigate promising targets and related metabolic pathways. This research provides scientific evidence for the combination of DSW and fucoidan (CDF) in the therapy for T2DM and other metabolic diseases.

## 2. Results

### 2.1. H-CDF Results in Better Improvement in Blood Glucolipid-Related Indices in T2DM Rats

**Figure 1:**
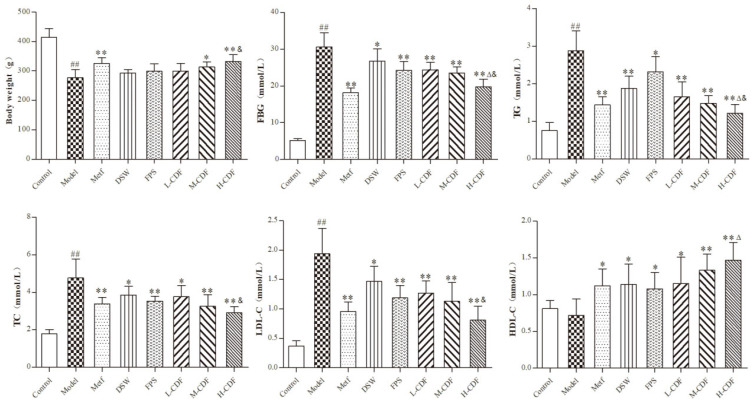
High-dose CDF treatment (H-CDF) resulted in better improvement in glycolipid indexes in T2DM rats compared to that with other doses (*n* = 8 for each group). L-CDF, M-CDF, and H-CDF are the low-, middle-, and high-dose CDF treatment groups, respectively. The administration of each group is detailed in Table 1. Error bars are expressed as means ± SDs, and statistical differences between groups were based on one-way ANOVA with Tukey tests for multiple-group comparisons and indicated using the following symbols: ^##^ *p* < 0.01 vs. control group; * *p* < 0.05, ** *p* < 0.01 vs. model group. ^Δ^ *p* < 0.05 vs. fucoidan (FPS) group; ^&^ *p* < 0.01 vs. DSW group.

The results of body weight and serum glucolipid-related indices in rats in all groups are displayed in Figure 1. A comparison between the normal group and model group showed that HFD/STZ treatment significantly reduced the body weights of rats in the model group, while levels of FBG, TC, TG, and LDL-C dramatically increased (*p* < 0.01). No significant differences were found in the contents of HDL-C. Nevertheless, the administration of DSW or FPS remarkably lowered the contents of FBG, TC, TG, and LDL-C (*p* < 0.05 or *p* < 0.01) and prominently raised HDL-C levels (*p* < 0.05 or *p* < 0.01). In addition, CDF treatment resulted in better outcomes than DSW or FPS alone in regulating glycolipid metabolism. In particular, a high dose of CDF (H-CDF) exerted the best effects.

### 2.2. H-CDF Preferably Improves Histopathological Changes and Akt/GSK3β Signaling in T2DM Rats

**Figure 2:**
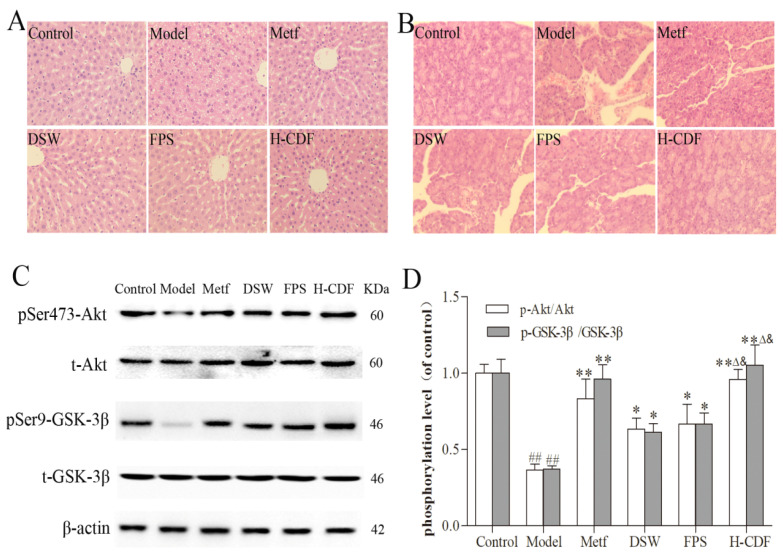
H-CDF alleviated liver and pancreas pathology, as well as the AKT–GSK signaling conduction defect, in T2DM rats (*n* = 8 for each group). Histopathological observations of the liver and pancreas are shown in (**A**,**B**), which were taken at 400× magnification. (**C**) Levels of total and phosphorylated Akt and GSK-3β in the liver were visualized using Western blotting (WB). (**D**) Levels were quantified based on three independent experiments. Statistical differences between groups were based on one-way ANOVA with Tukey tests and indicated using the following symbols: ^##^ *p* < 0.01 vs. control group; * *p* < 0.05, ** *p* < 0.01 vs. model group. ^Δ^ *p* < 0.05 vs. fucoidan group; ^&^ *p* < 0.01 vs. DSW group.

Pathological sections of the livers in each group are shown in Figure 2A. Severe steatosis was distinctly observed in hepatocytes of the model group. In contrast, DSW and fucoidan reduced lipid accumulation in liver cells and mitigated liver damage in T2DM rats. Pancreatic pathology was also alleviated after the administration of DSW or fucoidan (Figure 2B).

The Akt/GSK-3β pathway is an important step in insulin signaling transduction, and the abnormality of this pathway is the basic mechanism of T2DM [19]. Therefore, we also monitored the expression of this signaling in the liver via WB. The protein expression of Akt and GSK-3β in these groups was not significantly different; however, HFD and STZ injection dramatically decreased the phosphorylation levels of Akt and GSK-3β in the model group, suggesting that the insulin signaling pathway was inhibited or damaged. Notably, DSW or fucoidan treatment also enhanced the phosphorylation of Akt and GSK-3β, indicating that DSW and fucoidan can effectively improve insulin signaling (Figure 2C,D). Original Western blots are shown in Supplementary Figure S1.

Histological examination and WB results both indicated that H-CDF exerted better effects than DSW or fucoidan alone. These results are consistent with the data of the biochemical assay and further verified the combined effects of DSW and fucoidan. Therefore, we explored the mechanism of H-CDF in the following experiments.

### 2.3. H-CDF Improves Metabolic Disorders in T2DM Rats

**Figure 3:**
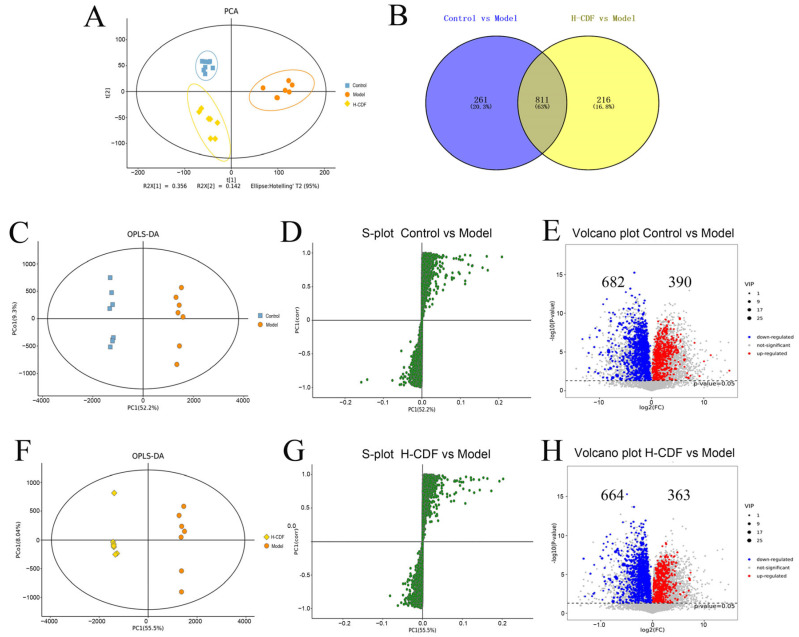
H-CDF improved metabolic disorders in T2DM rats (*n* = 7–8 for each group), as shown by multivariate analysis. (**A**) PCA score plot of each group. (**B**) Venn diagram for the pairwise comparisons. (**C**–**E**) OPLS-DA score scatter plots, S-plot, and volcano plot between control group and model group. (**F**–**H**) OPLS-DA score scatter plots, S-plot, and volcano plot between model group and H-CDF group. (**A**) was analyzed via PCA, (**C**,**F**) were analyzed via OPLS-DA, (**E**,**H**) were carried out by performing a Student’s t test and fold-change analysis.

Base-peak ion (BPI) diagrams of fecal samples from the control, model, and H-CDF groups are shown in Supplementary Figure S2. The metabolic profiles of these three groups were assayed using principal component analysis (PCA). From Figure 3A, the control and model groups were relatively far from one another and notably distinguished, suggesting that under the induction of HFD and STZ injection, body metabolism of the model rats was greatly disturbed. In contrast, the H-CDF group showed a tendency to come closer to the control group, indicating that the metabolic disorder in T2DM rats had been alleviated to a certain extent after H-CDF intervention.

To maximize the differences among these groups, supervised orthogonal partial least-squares discriminant analysis (OPLS-DA) was carried out. Figure 3C,F show that the control group and H-CDF group were notably different from the model group on the OPLS-DA score map. Then, an S-plot was applied to identify the potential biomarkers based on the variable importance in the projection (VIP) value. As shown in Figure 3D,G, each green circle represented a metabolite, and points far from the origin represented metabolites with larger VIP values that enable distinction from other groups. Volcano plots (Figure 3E,H) show that 1072 differential metabolites were found between the control group and model group, while 1027 differential metabolites were picked out between the H-CDF group and model group. Notably, 63% of the differential metabolites (811 in total) were shared by the two pairs, illustrating that H-CDF improved the metabolic disorder of T2DM.

### 2.4. Identification of Potential Biomarkers and Analysis of Pathway Enrichment for Effect of H-CDF in Improving Insulin Resistance

**Figure 4:**
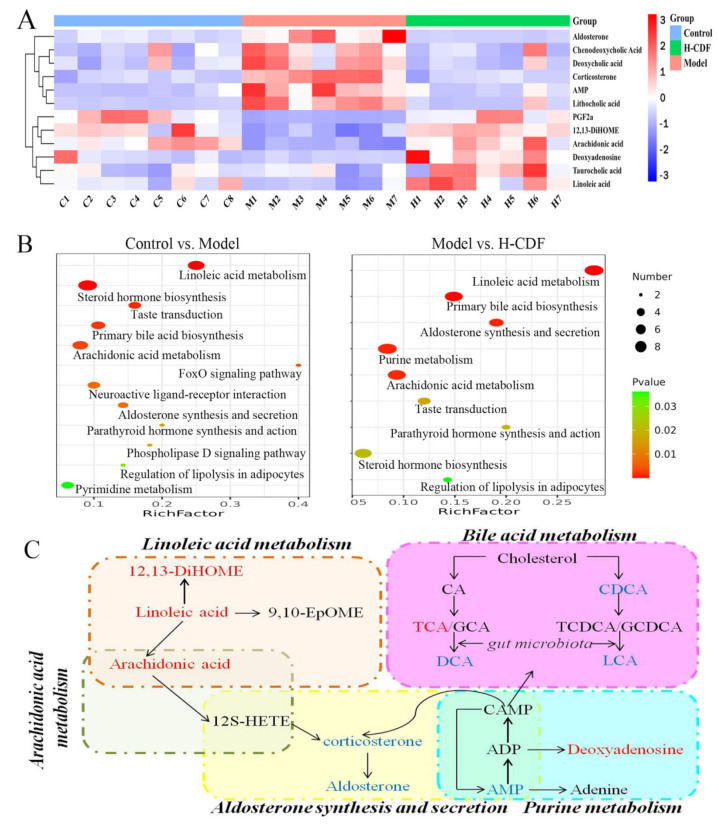
Differential metabolites and analysis of pathway enrichment of H-CDF for improving insulin resistance. (**A**) Heatmap of levels of nine metabolites in these three groups (*n* = 7–8 rats for each group). These metabolites were screened out using OPLS-DA combined with a Student’s t test. (**B**) The metabolomic pathways involved in T2DM and H-CDF intervention were explored using Metaboanalyst 5.0 (*p* < 0.05). (**C**) Network map associated with differential metabolic pathways in the H-CDF group. Words in red font indicate metabolites that prominently increased, while words in blue font indicate metabolites that were downregulated.

Screening of potential biomarkers was carried out based on the standard of VIP > 1 and *p* < 0.05 (using t-tests). The screening results, combined with database retrieval (HMDB) and a literature comparison, resulted in 12 differential metabolites, as shown in Table 2 and Figure 4A. The metabolites included nine compounds in positive ion mode and three in negative ion mode. Specific information on these compounds is displayed in Table 2. In the feces of model rats, five metabolites, including arachidonic acid, deoxyadenosine, linoleic acid (LA), 12,13-DiHOME, and taurocholic acid (TCA), were significantly downregulated, and six metabolites, including chenodeoxycholic acid (CDCA), deoxycholic acid (DCA), corticosterone, AMP, LA, and aldosterone, were significantly upregulated. H-CDF intervention could significantly regulate these differential metabolites. Visualization of these 12 potential biomarkers via heatmap analysis shows that H-CDF had a good adjustment effect on these metabolites (Figure 4C).

Pathway enrichment of diabetes-related biomarkers was conducted using Metaboanalyst 5.0. In total, 11 metabolic pathways were found between the control and model groups, mainly involving LA metabolism, primary bile acid (BA) biosynthesis, steroid hormone biosynthesis, taste transduction, and arachidonic acid metabolism. H-CDF significantly improved nine of the 12 signaling pathways, especially LA metabolism, primary BAs biosynthesis, aldosterone synthesis and secretion, arachidonic acid metabolism, and purine metabolism. Based on these results, we drew an association map of differential metabolic pathways in Figure 4C. The metabolic pathway enrichment results of the H-CDF group are shown in Supplementary Table S1.

DCA and lithocholic acid (LCA) are typical secondary BAs formed through the action of gut microbes. H-CDF showed good regulatory effects on BA metabolism; therefore, the ability of H-CDF to mitigate T2DM may be closely related to gut microbiota. Based on this finding, we conducted 16S rDNA analysis.

### 2.5. H-CDF Improves Bacterial Diversity in T2DM Rats

**Figure 5:**
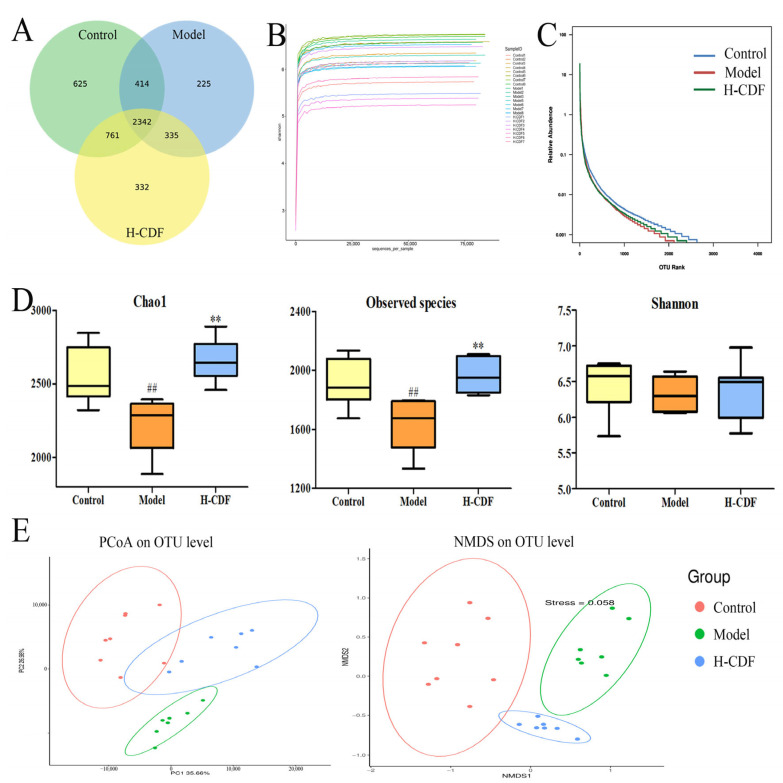
Diversity analysis of intestinal flora (*n* = 7–8 rats for each group). (**A**) Venn diagram; (**B**) dilution curve; (**C**) rank abundance curve; (**D**) alpha diversity analysis consisted of chao 1, observed species, and the Shannon index. A Wilcoxon rank sum test was applied for pairwise comparisons, and statistical differences shown are indicated using the following symbols: ^##^ *p* < 0.01 vs. control group; ** *p* < 0.01 vs. model group. (**E**) Beta diversity analysis includes PCoA and NMDS analysis, which were analyzed via PCOA or NMDS based on the Bray Curtis distance matrix algorithm.

In 16S rDNA analysis, sequences after quality-control were classified into multiple operational taxonomic units (OTUs) at 97% similarity using Vsearch. From Figure 5A, the OTU number in the H-CDF group increased to 3790, which was 454 more than that in the model group. Moreover, H-CDF shared 3103 OTUs with the control group, while it only shared 2677 OTUs with the model group.

Alpha diversity is a universal indicator for the richness and diversity of a microbial community. Chao1, observed species, and Shannon indices, shown in Figure 5D, illustrated that the abundance and diversity of gut microbiota were reduced in T2DM rats, and the administration of H-CDF helped to increase these indicators to normal levels. Rarefaction curve and rank abundance (Figure 5B,C) indicated that the information on the samples was reasonable and large enough for diversity assay.

Principal co-ordinate analysis (PCoA) and nonmetric multidimensional scaling (NMDS) analysis (Figure 5D) indicated that the intestinal flora of these three groups were clustered into three categories. The control and model groups were distinctly separated from each other and located on the far sides of the two-dimensional map, respectively, indicating that T2DM resulted in noteworthy changes in the gut microbiota of rats. The H-CDF group was close to the control group, suggesting that H-CDF could regulate the flora of diabetic rats and restore it to normal.

### 2.6. H-CDF Regulates the Structure of the Intestinal Flora at Multiple Taxonomic Levels

**Figure 6:**
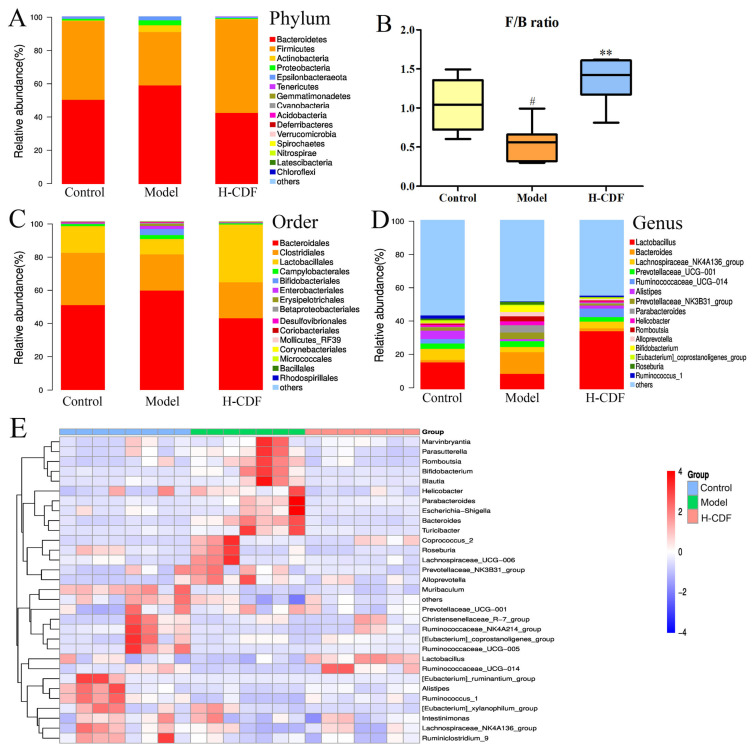
Community structure and heatmap analysis. (**A**,**B**) show relative abundances of the top 15 phyla and the F/B ratio in each group (*n* = 7–8 rats for each group); (**C**,**D**) display relative abundances of the top 15 differential species at the order and genus levels. Heatmap analysis in (**E**) represents the abundances of the top 30 genera, which are indicated using a red-to-blue pattern (from high to low) and variation tendency in the three groups through a genus-based comparison of the intestinal flora. Differential species among the three groups were analyzed by performing the Kruskal–Wallis test, and statistical differences shown in B are indicated using the following symbols: ^#^ *p* < 0.05 vs. control group; ** *p* < 0.01 vs. model group.

The flora composition of each group was assessed at the phylum, order, and genus levels. Data on the phylum level are depicted in Figure 6A. Dominant microflora in all three groups were Firmicutes and Bacteroidetes (over 90%). The abundance of Firmicutes in the model group was reduced significantly, compared with that in the control rats. However, abundances of Bacteroidetes and Actinobacteria notably increased, and the F/B ratio significantly increased. After H-CDF intervention, the abundance of Firmicutes increased to normal levels, and the F/B ratio increased significantly (Figure 6B).

At the order level (Figure 6C), Bacteroidales was markedly enriched, and Clostridiales and Lactobacillales were reduced sharply in T2DM rats. H-CDF reversed this change nicely.

As depicted in Figure 6D, the genera with relatively high abundance in the normal rat microbiota were *Lactobacillus* (15.76%), *Lachnospiraceae NK4A136 group* (6.61%), and *Bacteroides* (1.40%). However, in rats of the model group, the abundance of *Lactobacillus* decreased, while *Bacteroides* increased significantly. H-CDF administration significantly enriched *Lactobacillus*, while *Bacteroidetes* decreased to near normal levels (2.50%).

A heat map (Figure 6E) was used to visualize the bacterial composition, which suggested that fucoidan significantly decreased the levels of *Romboutsia*, *Bacteroides*, and *Parabacteroides* and significantly enriched *Lactobacillus* and *Ruminococcaceae UCG-014*.

**Figure 7:**
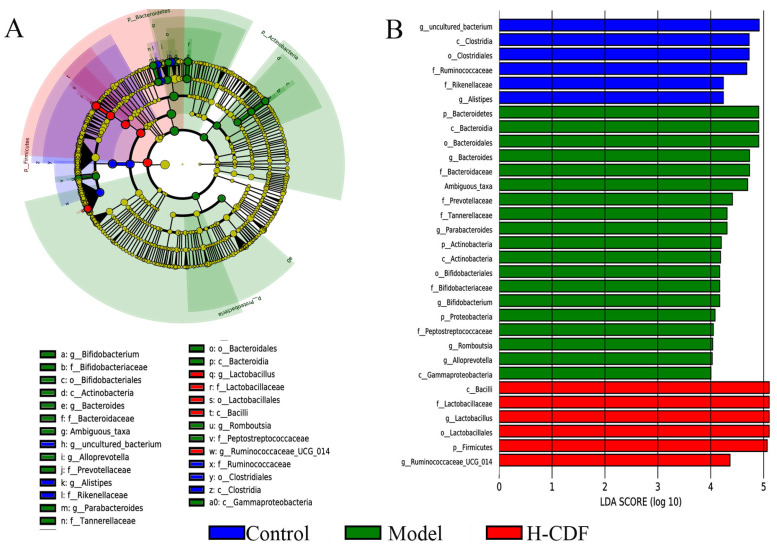
LEfSe cladogram (**A**) and histogram of LDA value distribution in each group (**B**) (*n* = 7–8 rats for each group) (LDA > 3). Letters before names of bacteria indicate taxonomic levels, i.e., p indicates phylum, c indicates class, o indicates order, f indicates family, and g indicates genus. Marker species depicted in A and B were screened via LEfSe.

Linear discriminant analysis effect size (LEfSe) was conducted to reveal potential marker species in each group. From Figure 7, the prominent difference was observed in the flora composition of these three groups at multiple levels from the phylum to genus. Significantly enriched communities in the control group included Clostridia, Clostridiales, and *Ruminococcaceae*. The different species in the model group were mainly Bacteroidetes, Bacteroidia, Bacteroidales, Bacteroidaceae, and *Bacteroides*. The H-CDF group was significantly enriched in Firmicutes, Bacilli, Lactobacillales, Lactobacillaceae, *Lactobacillus*, and *Ruminococcaceae UCG-014*. These data suggest H-CDF could significantly enrich *Lactobacillus* and *Ruminococcaceae UCG-014* and inhibit *Bacteroides* to improve the intestinal flora in T2DM rats.

### 2.7. Spearman Correlation Analysis between Differential Flora and Metabolites in H-CFD Group

**Figure 8:**
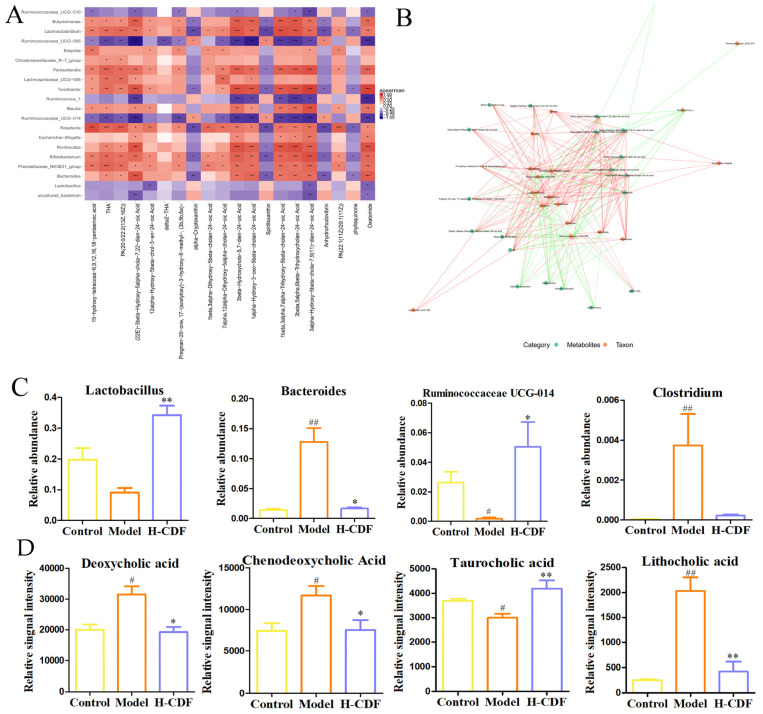
Correlation assay between differential metabolites and intestinal flora. (**A**) Heat map visualizing the correlation between differential flora and metabolites. Positive and negative correlations are represented using a red-to-blue pattern. Deeper colors represent closer correlations. The correlation between significantly differential OTUs and metabolites was analyzed using Spearman’s correlation analysis. * *p* < 0.05, ** *p* < 0.01 and *** *p* < 0.001. (**B**) Correlation network diagram. The red or green line indicates a positive or negative correlation, respectively, and the thickness of the lines represents levels of the correlation coefficient. (**C**) Relative abundance of four bacterial groups. (**D**) Relative content of four BAs. Differential species shown in (**C**,**D**) were analyzed via the Kruskal–Wallis test, and statistical differences shown are indicated using the following symbols: ^#^ *p* < 0.05, ^##^ *p* < 0.01 vs. control group; * *p* < 0.05, ** *p* < 0.01 vs. model group.

Spearman correlation analysis was carried out between differential genera and metabolites (Top20-20) in the H-CDF group (Figure 8). Ten of the top 20 differential metabolites belonged to BAs and derivatives, which suggests that the gut microbiota–BA interaction has an essential function in the beneficial effects of H-CDF on T2DM rats. *Ruminococcaceae* and *Lactobacillus* were negatively correlated with the contents of BA derivatives, while *Bacteroides* were positively correlated with them. Many studies have shown that BA metabolism is strongly correlated with the intestinal microecology, and primary BAs need to undergo a deconjugation reaction (i.e., to remove glycine or taurine conjugates) to be converted into secondary BAs in the intestine [20,21]. The BA deconjugation process is dominated by bacteria that have bile salt hydrolase activity, which mainly include *Lactobacillus*, *Bacteroides*, *Ruminococcaceae*, and *Clostridium* [22,23].

Therefore, we monitored the changes in these four strains in the three groups. As shown, H-CDF significantly enriched probiotics, such as *Lactobacillus* and *RuminococcaceaeUCG-014*, and significantly decreased the levels of *Bacteroides* and *Clostridium*. These microbiota changes led to the modulation of BA metabolism, as shown in Figure 8D. After H-CDF administration, the hepatotoxic hydrophobic secondary BAs, such as DCA and LCA, were greatly decreased. Simultaneously, the level of taurocholic acid (TCA), a more hydrophilic BA, increased, thereby reducing the toxicity of BAs and enhancing the dissolution of ingested lipids.

### 2.8. H-CDF Inhibits the FXR–FGF15 Pathway in Ileum Cells

**Figure 9:**
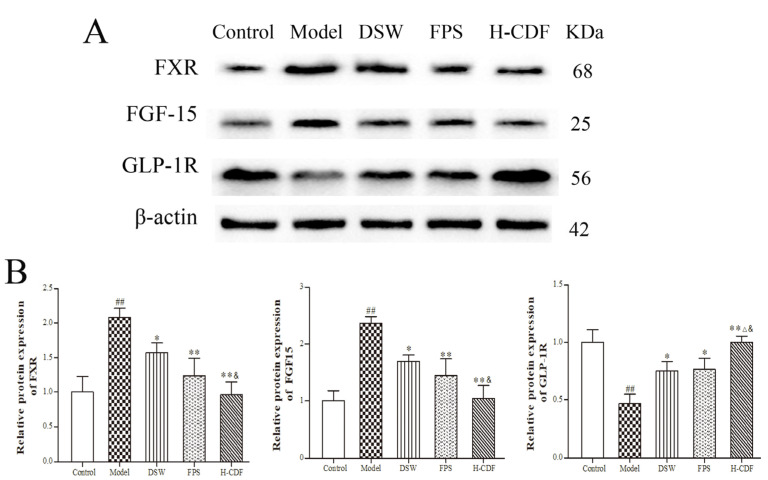
H-CDF administration inhibits the FXR–FGF15 pathway with stimulation of GLP-1R. Protein levels of FXR, FGF-15, and GLP-1R in the ileum were detected using WB. We repeated experiments three times, and blots displayed were the most representative (**A**); quantification of the blots is presented as the mean ± SD (*n* = 3 each group) (**B**). mRNA level of Statistical differences between groups were based on one-way ANOVA with Tukey tests and indicated using the following symbols: ^##^ *p* < 0.01 vs. control group; * *p* < 0.05, ** *p* < 0.01 vs. model group; ^&^ *p* < 0.05 vs. DSW group; ^Δ^ *p* < 0.05 vs. FPS group.

As an endocrine signaling molecule, BAs can regulate metabolic processes after binding to the receptors FXR and TGR5, among which CDCA, DCA, and LCA are the main agonists of FXR [24]. Notably, the levels of CDCA, DCA, and LCA in these groups were quite different. Thus, we performed WB to detect protein levels of FXR and its downstream effectors, such as FGF15 and glucagon-like peptide-1 receptors (GLP-1R) in the ileum. The data in Figure 9 show that the protein contents of FXR and FGF-15 were markedly increased in T2DM rats; however, the levels of GLP-1R were notably decreased. The data suggest that the FXR pathway was abnormally activated under the induction of HFD, which is in accordance with Huang’s research results [25]. H-CDF administration can significantly reverse these changes, including the inhibition of FXR and FGF-15 and activation of GLP-1R. Original Western blots are shown in Supplementary Figure S3.

## 3. Discussion

In this research, a high-dose combination of DSW and fucoidan (H-CDF) mitigated T2DM symptoms in rats through the modulation of glycolipid levels, alleviation of hepatopancreatic pathology, and activation of the Akt/GSK-3β pathway. Fecal-based metabolomics analysis showed that H-CDF intervention notably modulated the metabolic pattern of rats, with many metabolites returning to normal levels. Moreover, 12 metabolites closely related to T2DM were identified, including arachidonic acid, LA, CDCA, DCA, and corticosterone. H-CDF intervention had a significant adjustment effect on 12 metabolites and significantly improved nine important pathways involving linoleic acid metabolism, primary BA biosynthesis, aldosterone synthesis and secretion, and arachidonic acid metabolism, which were strongly related to the therapeutic effects of H-CDF.

It has been reported that LA can accelerate intracellular fatty acid oxidation, promote the decomposition of glucose, and significantly reduce the incidence of T2DM [26,27]. As a metabolite of linoleic acid, 12,13-diHOME signals brown adipocytes to burn lipids, and its plasma level is inversely associated with obesity [28]. BAs have essential functions in modulating metabolism, especially concerning lipids, glucose, and energy [29]. Among them, hydrophobic BAs, such as CDCA, DCA, and LCA, are known as toxic BAs, as they can cause damage, necrosis, and apoptosis in liver cells. For example, DCA can cause an inflammatory response and endoplasmic reticulum stress, followed by impaired glucose regulation and insulin resistance [30]. Studies have shown that excessive aldosterone can lead to metabolic disturbances, involving glucose metabolism disorders, insulin resistance and the consequent development of T2DM, impaired lipid metabolism, and metabolic syndromes [31]. Arachidonic acid can promote the β-oxidation of fat in the body, which can effectively reduce the content of blood lipids and LDL-C, as well as prevent the accumulation of lipids in the blood [32]. As shown in Figure 4C, there is a close connection between these pathways; H-CDF can comprehensively regulate these metabolic pathways and improve glucose–lipid metabolism disorders.

Of note, a growing body of research has indicated that BA metabolism depends on intestinal microecology. In addition, the intestinal flora can participate in host metabolic processes of carbohydrates, BAs, and short-chain fatty acids, thereby mediating the occurrence and progress of metabolic diseases, especially T2DM [33].

A comparison of gut microbials based on 16S rDNA revealed that the richness and diversity of the gut flora in T2DM rats decreased significantly. However, H-CDF intervention significantly increased the richness and diversity of intestinal flora. As it concerns species composition, the results show that the phylum Bacteroidetes was notably enriched in T2DM rats; nevertheless, the phylum Firmicutes was dramatically reduced. H-CDF treatment significantly reversed these changes and increased the ratio of F/B. Larsen et al. [34] and Zhao et al. [35] showed that the blood glucose level has a negative correlation with F/B. Thus, increasing the F/B ratio can ameliorate the intestinal microecology and lower glucose levels in individuals with T2DM [36,37], and this finding is consistent with our results.

At the genus level, T2DM rats showed notably enriched *Bacteroides*, but the abundances of *Lactobacillus* were drastically reduced. This is consistent with the research of Kanazawa et al. [38], in which the abundance of *Lactobacillus* in a T2DM population was lower than that in the normal population. *Lactobacillus* supplementation showed various health benefits for T2DM in a double-blind randomized controlled trial [39]. Yan et al. [40] found that *Lactobacillus* supplementation modulated the levels of genes that participate in glycolipid metabolism and improved the epithelial barrier function in T2DM. In our study, H-CDF significantly enriched the abundance of *Lactobacillus*, which was verified both in heatmap and LEfSe analyses. Moreover, *Ruminococcaceae UCG-014* was a distinguished species in the H-CDF group, which has been claimed to be inversely associated with metabolic disturbances and the pathogenesis of diabetes [41]. This suggests that the regulatory effect of H-CDF on *Lactobacillus* and *Ruminococcaceae UCG-014* may be strongly associated with its hypoglycemic activity.

Interestingly, *Lactobacillus*, *Ruminococcus*, and *Bacteroides* mainly take part in the deconjugation of primary BAs, and it has been reported that an intestinal flora imbalance can activate FXR by affecting intestinal BA metabolism [42], increasing the expression of FGF15, reducing hepatic BA synthesis, and causing cholesterol metabolism disorders [43]. In contrast, the inhibition of FXR can promote GLP-1 production and secretion, thereby activating GLP-1R, enhancing insulin sensitivity, and effectively regulating blood glucose homeostasis [44].

**Figure 10:**
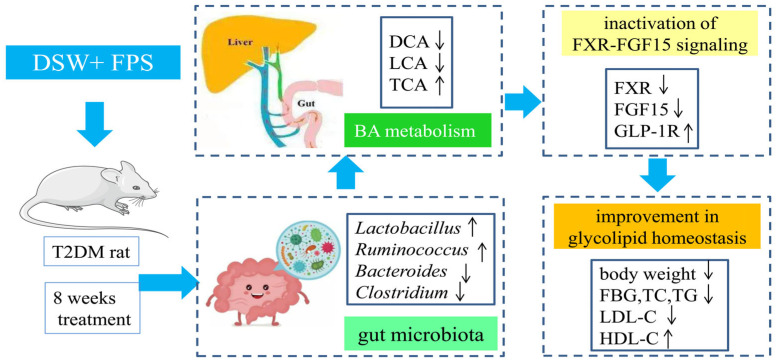
Mechanistic diagram of effects of H-CDF on T2DM. H-CDF enriched *Lactobacillaceae* and *Ruminococcaceae UCG-014*, thereby changing BA metabolism, as well as the inactivation of FXR–FGF15 signaling, and improving glucose and lipid metabolism.

In our study, the Spearman correlation between differential metabolites and intestinal flora (Top20-20) also indicated that the gut microbiota–BA interaction had an essential function in the mechanism of H-CDF in T2DM rats. Increased levels of harmful flora, for instance, *Bacteroides* and *Clostridium*, were observed in T2DM rats, while beneficial bacteria, such as *Lactobacillus* and *Ruminococcus,* decreased significantly. These data are consistent with the report by Gu’s team [45] on T2DM patients. However, H-CDF treatment significantly reversed the changes in these flora, thereby reducing the levels of DCA and LCA, which are toxic secondary BAs. The WB results showed that H-CDF could more significantly inhibit FXR–FGF15 signaling and increase the levels of GLP-1R, compared to those with DSW or fucoidan alone. Its main mechanism can be described as shown in Figure 10. 

Previous studies have shown that fucoidan is a high-quality polysaccharide prebiotic. Although it is rarely digested and absorbed in the gastrointestinal tract, it can provide a good carbon source for the intestinal flora to facilitate the proliferation of probiotics, ameliorate the microbial structure through the regulation of its metabolites, and thereby improve the intestinal barrier and T2DM [46,47]. Zhang et al. [48] showed that FPS can improve insulin resistance in HFD-induced mice, and the key differential bacteria were Bacteroidetes, Bacteroidales, and Alistipes. Wu’s research [49] indicated that fucoidan decreased the FBG levels in HFD/STZ-induced mice, which is related to the increase in F/B and enrichment of *Lactococcus lactis* and *Lachnoclostridium* spp.

DSW is rich in beneficial mineral elements, and studies have demonstrated that DSW can improve the intestinal flora to lower cholesterol in hamsters with hypercholesterolemia [50]. Moreover, it can significantly improve the intestinal environment of healthy adults, raise the level of SCFA in feces, and impart a wide range of beneficial effects in humans [51]. As the main component of DSW, Mg significantly improved the diversity of the gut microbiota in diabetic mice, increased Firmicutes and decreased Bacteroidetes abundance, and significantly enriched *Lactobacillales*, in addition, Mg can also ca regulate bile acid metabolism [35]. Petrič et al. found that Zn could increase the abundance of *Ruminococcus albus* [52]. Ferreira’s review concluded that Se was able to balance the microbial flora, avoiding health damage associated with dysbiosis [53].

Here, we found that the combination of DSW and fucoidan modulated the intestinal flora, especially the enrichment of *Lactobacillaceae* and *Ruminococcaceae UCG 014*, and decreased the levels of *Bacteroides*, which should be the synergistic effects of fucoidan and minerals in DSW, thereby changing the BA metabolism profile, affecting the host BA signaling and metabolic pathways, such as LA and arachidonic acid metabolism, enhancing insulin sensitivity, and preferably improving glycolipid metabolism in T2DM rats. Our study offers a foundation for the combined use of DSW and fucoidan in therapy for T2DM.

## 4. Materials and Methods

### 4.1. Preparation and Elementary Analysis of DSW

The preparation and elemental detection of DSW were conducted as described in our previous research [54]. In DSW, the contents of Na, Ca, and Mg were 69.63, 68.41, and 202.12 mg·L^−1^, respectively. In the aspect of trace elements, levels of V, Cr, Zn, and Se were 0.64, 1.22, 4.17, and 0.51 µg·L^−1^, respectively. 

### 4.2. Analysis of Fucoidan Physicochemical Properties

Physicochemical properties of fucoidan were analyzed; the monosaccharide compositions were analyzed using gas chromatography (GC), and the contents of total carbohydrates and the sulfate group were measured via phenol-vitriolic colorimetry and the Barium chloride-gelatin turbidimetric method, respectively [55]. The contents of total carbohydrates and the sulfate group were 50.69% and 26.04%, respectively. Monosaccharide composition analysis showed that in fucoidan, the ratio of Rha-Ara-Fuc-Xyl-Man-Glc-Gal was 4.75:12.09:34.23:2.77:3.04:1.00:7.18, and the GC chromatogram of the monosaccharide composition is shown in Supplementary Figure S4.

### 4.3. T2DM Model Induction and Drug Administration in Rats

SD rats (130 ± 10 g, male, *n* = 90) were obtained from Weitong Lihua Animal Technology Co., Ltd. (Beijing, China) and kept in the Animal Center of Shandong University of Traditional Chinese Medicine. All rats were acclimated for 7 days, and then, they were divided into two groups according to the weight. Ten rats were treated with a standard diet and denoted as the control group, while the remaining 80 rats were given an HFD (Keaoxieli, Beijing, China). After four weeks of diet induction, all rats were fasted overnight. On the following day, rats receiving HFD were administered 1% STZ via intraperitoneal injection (Sigma, USA) dissolved in citrate buffer at a dose of 35 mg·kg^−1^, and rats in the control group received an injection of citrate buffer solution only. The FBG level was determined with a test strip 72 h later, and rats with FBG higher than 16.7 mmol·L^−1^ were placed in the T2DM model group [56]. Then, according to the FBG and body weight, we randomly divided the successful T2DM rats into seven groups as follows: model group, Metf (SASS, Shanghai, China) group, DSW group, fucoidan (Changlong Biochemical Pharmaceutical Co., Ltd., Jilin, China) group, low-dose combination group of DSW and fucoidan (L-CDF), middle-dose combination group (M-CDF), and high-dose combination group (H-CDF). Each group was composed of 8 rats. The feeding and administration schedule of each group is shown in Table 1 and lasted for four weeks.

On the last day of administration, fresh feces were collected using a metabolic cage. All rats were fasted overnight and sacrificed the following day. Serum, liver, pancreas, and ileum samples were collected for further detection.

### 4.4. Biomedical Analysis

The FBG level in each group was monitored using a glucose meter (ACCU-CHEK performa, Roche, Basel, Switzerland). Final biochemical indicators, such as FBG, TC, TG, HDL-C, and LDL-C, were monitored on a URIT-8026 automatic biochemical analyzer (Urit Inc., Guilin, China).

### 4.5. Histological Analysis

The pancreas and left liver samples were prepared for histological studies; they were maintained in 10% formaldehyde solution and imbedded in paraffin. The paraffin blocks were cut into 5 µm slices and dyed in hematoxylin-eosin (HE). Thereafter, histopathology in the tissues was viewed using a light microscope (Olympus, Tokyo, Japan) [57]. The right liver lobes were kept at −80 °C until analysis.

### 4.6. Untargeted Metabolomics Analyses

Sixty milligrams of fecal samples from each rat were mixed with 600 μL of extraction solvent (cold methanol: water 4:1, *v*/*v*). 2-Chlorophenylalanine (Hengchuang Bio-technology Co., Ltd., Shanghai, China), which was used as the internal standard, was dissolved in methanol to prepare a 0.3 mg/mL solution. Twenty microliters of the internal standard solution was also put in the samples, followed by ultrasonic extraction and centrifugation at 13,000 rpm for 10 min at 4 °C. Then, the supernatant was dried using a freeze dryer and reconstituted in 400 μL of extraction solvent, followed by a second centrifugation. The supernatant was filtered and transferred to LC injection vials for HPLC analysis. Equal volumes of extracts from all samples were mixed to obtain a quality control sample [18,58].

Metabolites were isolated on an ACQUITY UPLC HSS T3 C18 column (100 mm × 2.1 mm, 1.8 μm) (Waters Corp, Milford, CT, USA) using an Exion LC system (AB Sciex, USA), and mass spectrometry (MS) was conducted on an AB Triple TOF 6600 plus system (AB Sciex, Framingham, MA, USA). For HPLC, mobile phase A was water acidified with 0.1% formic acid (*v*/*v*), and mobile phase B was acetonitrile acidified with 0.1% formic acid (*v*/*v*). The flow rate was 0.35 mL min^−1^, and the optimized elution gradient was as follows: 0 to 2 min, 5% B; 2 to 4 min, 5% to 30% B; 4 to 8 min, 30% to 50% B; 8 to 10 min, 50% to 80% B; 10 to 14 min, 80% to 100% B; 14 to 15 min, 100% B. For ESI MS, the scanning range was from *m/z* 100 to 1000; the ion source temperature was 550 °C; the curtain gas was 35 PSI; the interface heater temperature was 550 °C; the ion spray voltage was 5500 V and 4500 V for positive and negative mode, respectively; and the declustering potential was optimized to 80 V and −80 V for the positive and negative mode, respectively. The solvents for this analysis, including methanol, acetonitrile, and formic acid, were obtained from CNW Technologies GmbH (Düsseldorf, Germany).

### 4.7. Identification of Potential Biomarkers and Metabolic Pathways

Data of LC-MS were obtained and processed using Progenesis QI (version 2.3, Waters Corp.). Then, we merged data from positive and negative modes to obtain more integrated information and carried out PCA and OPLS-DA to obtain a visual representation of the changes in metabolic profiles among the control, model, and H-CDF groups. Selection standards of VIP > 1 and *p* < 0.05 were applied to obtain differential metabolites, and metabolic pathway enrichment was executed using MetaboAnalyst (https://www.metaboanalyst.ca/ (accessed on 22 February 2022)) [59].

### 4.8. 16S rDNA Gene Sequencing

We extracted the DNA of the gut microbiota from fecal samples (7–8 rats per group) using the DNeasy PowerSoil Kit (QIAGEN, Dusseldorf, Germany) based on the instructions. Then, we assayed the concentration of DNA through agarose gel electrophoresis with a Nanodrop 2000 spectrophotometer (Thermo Fisher Scientific, Waltham, MA, USA) [60]. Afterwards, 343F/798R primers were employed for PCR amplification of the V3V4 region, which was performed under the following cycling parameters: preheat at 94 °C for 5 min, 26 cycles, which included denaturation at 94 °C for 30 s, annealing at 56 °C for 30 s, elongation at 72 °C for 20 s, and a final elongation at 72 °C for 5 min. Then, Ampure XP beads were used to purify PCR products. The purity of the products was detected using agarose gel electrophoresis, and the concentration was determined using a Qubit. DNA sequencing was performed to obtain 16S rDNA sequences.

For the sequencing assay, low-quality reads were removed using Trimmomatic. Paired-end sequences after decontamination were spliced using FLASH software, based on the overlap relationship, and then, Vsearch (version 2.4.2) was used to aggregate valid sequences into OTUs at 97% similarity for species classification. Based on the OTUs, alpha diversity was calculated to evaluate species diversity and richness. In addition, rarefaction and rank abundance curves were drawn to assess the reliability of the sequencing results [61]. Beta diversity was determined via PCoA and NMDS to evaluate the similarity of species diversity and community composition between groups. LEfSe analysis (LDA > 3) was conducted to visualize potential differential flora between groups [60].

### 4.9. Spearman Correlation Analysis

The correlation between significantly differential OTUs and metabolites was analyzed using Spearman’s correlation analysis. At the genus level, Spearman correlation coefficients between significant OTUs and metabolites were calculated and presented in the forms of heatmaps and association network graphs [62].

### 4.10. WB Detection

Right liver lobe and ileum samples were lysed in ice-cold RIPA buffer containing 1 mM PMSF and phosphatase inhibitor (Beyotime Technology, Shanghai, China). The lysis lasted for 30 min, and lysates were centrifugated at 14,000× *g* for 5 min at 4 °C. Supernatants were collected, followed by determination of protein concentrations using the BCA method. Protein extracts were denatured with loading buffer by heating for 10 min in boiling water. Then, 40 μg protein samples of each lysate were loaded and isolated on 10% or 12% sodium dodecyl sulfate polyacrylamide gels, followed by transfer to PVDF membranes (Millipore Corporation, Tullagreen, IRL). The membrane was blocked using TBST solution containing 5% BSA (Solarbio Technology, Beijing, China) for 2 h at room temperature; then, it was incubated with a primary antibody (anti-GSK-3β, 1:1000, CST; anti-Phospho-GSK-3 (Ser9), 1:500, CST; anti-Akt, 1:8000, CST; anti-Phospho-Akt (Ser473), 1:500, CST; anti-FXR, 1:500, Abcam; anti-FGF15, 1:1000, Santa Cruz Biotechnology; anti-GLP-1R, 1:1000, Abcam) overnight at 4 °C. Thereafter, we used TBST solution to wash the membrane three times and incubated the membrane with anti-mouse or anti-rabbit IgG conjugated with horseradish peroxidase for 60 min. ECL reagents (Proteintech, Wuhan, China) were used to visualize the bands through a gel imaging system (Tanon 4800 Mu, Tanon Science & Technology Co., Shanghai, China). Finally, we used Image J to quantify protein expression by calculating the grayscale of the bands and normalizing the data to the grayscale of β-actin [25].

### 4.11. Statistical Analysis

Data analysis was performed with Graphpad. The data are shown in the form of mean ± SD, and the statistical methods applied included one way ANOVA, Student’s t-test, Kruskal–Wallis test, LEfSe, and Spearman’s correlation analysis. The detailed statistical processing is illustrated in the figure legend.

## 5. Conclusions

Overall, the orally administered combination of DSW and fucoidan exhibits better efficacy in alleviating T2DM symptoms than DSW or fucoidan alone. The mechanism is closely related to its enrichment of *Lactobacillaceae* and *Ruminococcaceae UCG-014*, thereby changing BA metabolism, LA metabolism, arachidonic acid metabolism, and other related pathways, as well as enhancing insulin sensitivity and improving glucose and lipid metabolism.

## 6. Strengths and Limitations of This Study

This study revealed that, for the first time, the orally administered combination of DSW and FPS can modulate metabolomics and the gut microbiota, thus exhibiting better efficacy in alleviating symptoms of T2DM than DSW or Fucoidan alone, providing inspiration for the development and utilization of the two marine resources. However, the object of our study is a mixture of DSW and fucoidan, and active ingredients of DSW and fucoidan are mineral elements and polysaccharides, respectively. There are still gaps in which the ingredient is directly effective for T2DM, which needs further exploration.

## Figures and Tables

**Table 1:** Feeding and administration in each group.

Groups	Drinking Water	Intragastric (ig) Administration	Dose/mg·kg^−1^ (ig)
Control	distilled water	distilled water	—
Model	distilled water	distilled water	—
Metf	distilled water	Metf	150
FPS	distilled water	fucoidan	70
DSW	DSW	distilled water	—
L-CDF	DSW	fucoidan	17.5
M-CDF	DSW	fucoidan	35
H-CDF	DSW	fucoidan	70

**Table 2:** Detailed information on significantly changed metabolites in the H-CDF group.

Metabolites	*m*/*z*	Rt (min)	VIP	Ion Mode	Formula	Change Trend		Fold-Change H/M
						M/C	H/M	
PGF2a	377.227	4.7717	1.68222	pos	C20H34O5	down	up	4.87625
Deoxyadenosine	252.109	2.20938	2.36237	pos	C10H13N5O3	down	up	3.72648
Linoleic acid	281.247	11.3704	5.63973	pos	C18H32O2	down	up	2.65520
Arachidonic acid	327.227	7.29902	2.01829	pos	C20H32O2	down	up	2.32247
12,13-DiHOME	313.238	10.3238	2.95125	neg	C18H34O4	down	up	1.903507
Taurocholic acid	516.301	7.08687	1.51054	pos	C26H45NO7S	down	up	1.27752
Chenodeoxycholic Acid	375.289	10.0277	3.27826	pos	C24H40O4	up	down	0.64045
Deoxycholic acid	410.325	10.6713	6.28420	pos	C24H40O4	up	down	0.61063
Corticosterone	385.175	4.55788	2.84140	pos	C21H30O4	up	down	0.30670
AMP	348.07	1.19063	2.49771	pos	C10H14N5O7P	up	down	0.25528
Lithocholic acid	375.246	9.58685	2.37074	neg	C24H40O3	up	down	0.20711
Aldosterone	359.189	8.7599	4.07483	neg	C21H28O5	up	down	0.11069

Note: metabolites were screened out using OPLS-DA combined with a Student’s *t* test.

## Data Availability

Data are contained within the article.

## Supplementary Materials

The following supporting information can be downloaded at: https://www.mdpi.com/article/10.3390/ph16030462/s1, Figure S1: GC chromatogram of monosaccharide composition in fucoidan; Figure S2: Western blot raw data for the Akt/GSK-3β pathway; Figure S3: BPI diagrams of each group in positive and negative ion mode; Figure S4: Western blot raw data for the FXR–FGF15β pathway; Table S1: Metabolic pathway enrichment results.
