# Supplementary material for: A Combination of Deep-Sea Water and Fucoidan Alleviates T2DM through Modulation of Gut Microbiota and Metabolic Pathways
Source: Pharmaceuticals (Basel). 2023 Mar 20;16(3):462. doi: 10.3390/ph16030462 (PMC10053583; doi:10.3390/ph16030462)
Supplement: Supplementary file 1 [file pharmaceuticals-16-00462-s001.zip › pharmaceuticals-2246648-supplementary.pdf]

Supplementary Material

1. Supplementary Figures

1.1. Western Blot Raw Data for Akt/GSK-3β Pathway

Original western blots for Akt/GSK-3β pathway are shown in Figure S2.

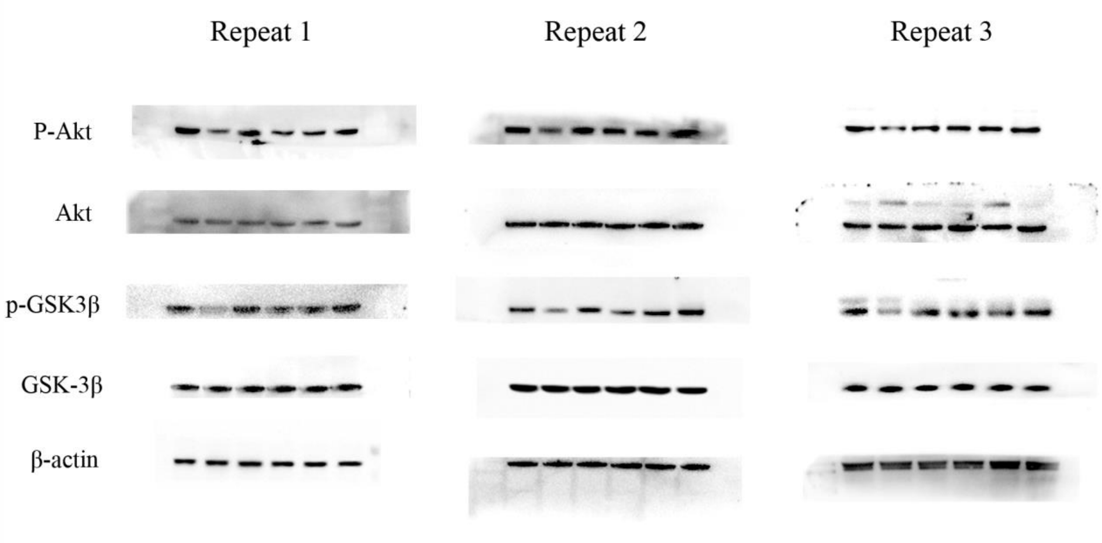

Figure S1. Original western blot for Akt/GSK-3β pathway.

1.2. BPI Diagrams of Each Group in Positive and Negative Ion Mode

Figure S3-A and B is the BPI diagrams of each group in positive and negative ion mode, respectively. We can see that, there were significant differences in endogenous metabolites between control and model groups, and the areas of chromatogram peaks at the same retention time were also different, indicating that high-fat diet significantly change the metabolic states of the rats, while H-CDF the H-CDF group was closer to the control group, suggesting the metabolic disorder in T2DM rats had been alleviated to a certain extent after H-CDF intervention

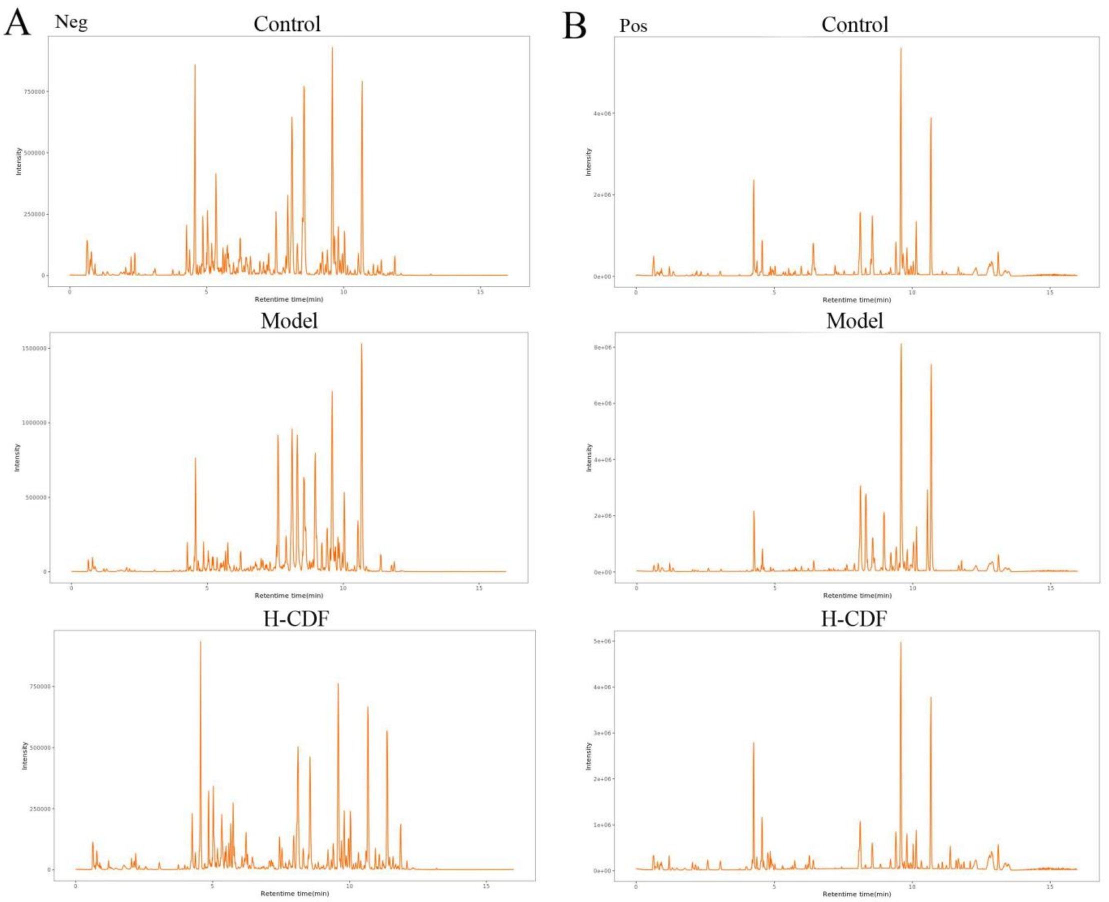

Figure S2. Representative BPI of fecal samples from rats in each group. A: Negative ion mode; B: Positive ion mode.

1.3. Western Blot Raw Data for FXR-FGF15 Pathway

Original western blots for FXR-FGF15 pathway are shown in Figure S4.

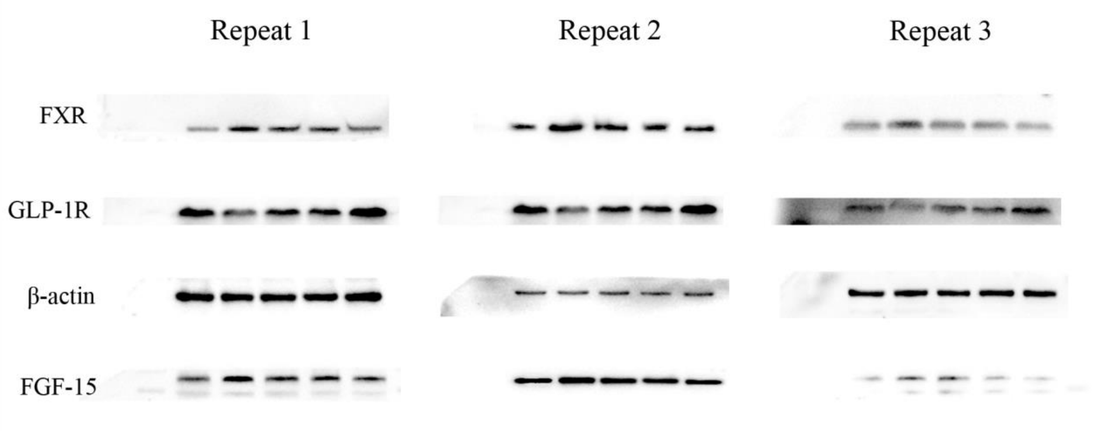

Figure S3. Original western blot for FXR-FGF15 pathway

1.4. GC Chromatogram of Monosaccharide Composition in Fucoidan

GC chromatogram of monosaccharide composition in fucoidan is shown in Supplementary Figure 1. The results

demonstrated that, ratio of Rha- Ara- Fuc- Xyl- Man- Glc- Gal is 4.75:12.09:34.23:2.77:3.04:1.00:7.18 in fucoidan.

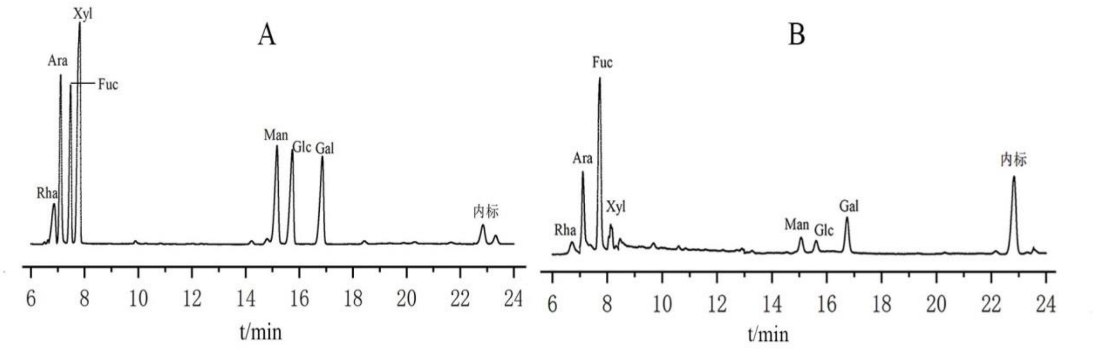

Figure S4. GC chromatogram of monosaccharide composition in fucoidan. A-Monosaccharide standards; B-Fucoidan.

2. Supplementary Tables

Metabolic pathway enrichment results of H-CDF group were shown in Table S1.

Table S1 Metabolic pathway enrichment results◎◎

Pathways	Hits	Total	Fold Enrichment	*p*-value	-lg (*p*-value)
Primary bile acid biosynthesis	8	28	0.2857	0.0000	7.0150
Linoleic acid metabolism	7	47	0.1489	0.0001	4.1743
Aldosterone synthesis and secretion	4	21	0.1905	0.0011	2.9758
Purine metabolism	8	95	0.0842	0.0011	2.9476
Arachidonic acid metabolism	7	75	0.0933	0.0013	2.8934
Taste transduction	3	25	0.1200	0.0176	1.7538
Parathyroid hormone synthesis, secretion and action	2	10	0.2000	0.0200	1.6990
Steroid hormone biosynthesis	6	99	0.0606	0.0227	1.6440
Regulation of lipolysis in adipocytes	2	14	0.1429	0.0382	1.4183
